# Characteristics of induced mutations in offspring derived from irradiated mouse spermatogonia and mature oocytes

**DOI:** 10.1038/s41598-019-56881-2

**Published:** 2020-01-08

**Authors:** Yasunari Satoh, Jun-ichi Asakawa, Mayumi Nishimura, Tony Kuo, Norio Shinkai, Harry M. Cullings, Yohei Minakuchi, Jun Sese, Atsushi Toyoda, Yoshiya Shimada, Nori Nakamura, Arikuni Uchimura

**Affiliations:** 1 Department of Molecular Biosciences, Radiation Effects Research Foundation, 5-2 Hijiyama Park, Minami-ku, Hiroshima 732-0815 Japan; 2 Department of Radiation Effects Research, National Institute of Radiological Sciences (NIRS), National Institutes for Quantum and Radiological Science and Technology (QST), Chiba, 263-8555 Japan; 3 Artificial Intelligence Research Center, AIST, 2-3-26 Aomi, Koto-ku, Tokyo 135-0064 Japan; 4 Real World Big-Data Computation Open Innovation Laboratory, AIST-Tokyo Tech, 2-12-1 Okayama, Meguro-ku, Tokyo 152-8550 Japan; 5 Department of Statistics, Radiation Effects Research Foundation, 5-2 Hijiyama Park, Minami-ku, Hiroshima 732-0815 Japan; 6 Comparative Genomics Laboratory, National Institute of Genetics, Mishima, 411-8540 Japan; 7 Humanome Lab, Inc., L-HUB 3F, 1-4, Shumomiyabi-cho, Sinjuku-ku, Tokyo 162-0822 Japan; 8 Department of Radiological Sciences, Graduate School of Human Health Sciences, Tokyo Metropolitan University, Tokyo, 116-8551 Japan; 9 Executive Director, QST, Chiba, 263-8555 Japan

**Keywords:** Next-generation sequencing, Risk factors

## Abstract

The exposure of germ cells to radiation introduces mutations in the genomes of offspring, and a previous whole-genome sequencing study indicated that the irradiation of mouse sperm induces insertions/deletions (indels) and multisite mutations (clustered single nucleotide variants and indels). However, the current knowledge on the mutation spectra is limited, and the effects of radiation exposure on germ cells at stages other than the sperm stage remain unknown. Here, we performed whole-genome sequencing experiments to investigate the exposure of spermatogonia and mature oocytes. We compared *de novo* mutations in a total of 24 F1 mice conceived before and after the irradiation of their parents. The results indicated that radiation exposure, 4 Gy of gamma rays, induced 9.6 indels and 2.5 multisite mutations in spermatogonia and 4.7 indels and 3.1 multisite mutations in mature oocytes in the autosomal regions of each F1 individual. Notably, we found two types of deletions, namely, small deletions (mainly 1~12 nucleotides) in non-repeat sequences, many of which showed microhomology at the breakpoint junction, and single-nucleotide deletions in mononucleotide repeat sequences. The results suggest that these deletions and multisite mutations could be a typical signature of mutations induced by parental irradiation in mammals.

## Introduction

The history of mutagenesis experiments began with exposure to ionizing radiation (IR) in 1926^1^, and since then, the mutagenic effects of IR have been studied extensively. IR induces various types of DNA damage ranging from nucleotide modifications to DNA strand breaks. Among the DNA damage induced by IR in germ cells, those that are not correctly repaired will lead to *de novo* mutations in the genomes of offspring^2^. The types and extent of mutations induced by IR in the mammalian germline have been a particularly important issue in the field of radiation biology. A substantial number of studies on human populations (the offspring of exposed individuals, such as atomic bomb survivors) have been conducted, but the heritable effects following exposure to IR have not been clearly elucidated^3–5^. Much of the decisive evidence has been obtained from studies using mouse models. In particular, screening experiments based on the visible phenotypes of the offspring of exposed parents, known as specific-locus tests, have provided important evidence for transgenerational effects. For example, the frequency of mutants increases with increases in the radiation dose (linear increases with the exposure of spermatogonia and sometimes exponential increases with the exposure of maturing and mature oocytes)^6^. The exposure of maturing and mature oocytes induces an approximately two-fold higher number of mutations than the exposure of spermatogonia^3,6^. However, because specific-locus tests have been limited to specific regions, typically seven protein-coding genes, it has been difficult to evaluate the effects on mutation induction at a genome-wide level.

The recent progress in high-throughput sequencing technologies enables us to perform whole-genome sequencing (WGS) and provides us with the chance to survey *de novo* mutations at the genome-wide level. One previous study using a WGS approach indicated that parental irradiation of 3 Gy at the sperm stage induces insertions/deletions (indels) and “multisite mutations”, clusters of single nucleotide variants (SNVs) and indels in close proximity, but not SNVs in the offspring^7^. However, the current knowledge on the mutation spectra is limited, and the genome-wide effects of IR on germ cells at stages other than the sperm stage have not been assessed using WGS techniques.

The heritable effects of IR are thought to differ depending on the developmental stage of gametogenesis at which the exposure occurs because these stages are characterized by significant differences in the chromosome conformation and the ability to repair DNA damage^8,9^. For example, the paternal and maternal gametogenesis processes are quite different, and a large portion of the genomic DNA in sperm is tightly packed with protamines instead of histones^10^. Indeed, specific-locus tests in mice have suggested that the effects on mutation induction in offspring show variations among different parental exposures: male germ cells that have entered meiosis, as well as maturing and mature oocytes, show increased sensitivity to IR than spermatogonia^3,11^. These data suggest that it is important to understand the effects of IR on germ cells other than those at the sperm stage.

In this study, we focused on the effects of IR on paternal germ cells at the spermatogonial stage and on maternal germ cells at the mature oocyte stage using a mouse model. The effects of IR on spermatogonia and immature oocytes are particularly important for assessing the risk of human exposure to IR. However, in this study, we investigated the effect on mature oocytes as a maternal exposure model. This stage was selected because immature mouse oocytes are sensitive to the lethal effect of IR, which makes it difficult to assess the effect on the genome^12^, and the exposure of mice to 0.45 Gy of radiation causes a severe loss of small primordial follicles from the ovary^13^. Here, we characterized and quantified the *de novo* mutations, mainly base substitutions and small indels, on spermatogonia and mature oocytes through WGS and consider the transgenerational effects of IR at the genome-wide level.

## Results

### Experimental design and detection of *de novo* mutations

It is known that 4 Gy of IR exposure leads to a substantial reduction of mouse spermatogonial cells and it takes 10 weeks or more for the recovery of reproductive capacity^14^, where it takes approximately 5 weeks for cells to develop from spermatogonia to sperm^8^. On the other hand, it is also known that 4 Gy of IR exposures have a killing effect on some portion of mature oocyte and reduce litter size of the offspring^6^. But, in our experiment, we found no apparent reduction of the litter size of the F1 mice born to the mother irradiated with 4 Gy of gamma rays (Supplementary Data 1). Therefore, we conducted two sets of experiments described below, to know the mutation-inducing effects of radiation exposure on spermatogonia and mature oocytes.

**Figure 1:**
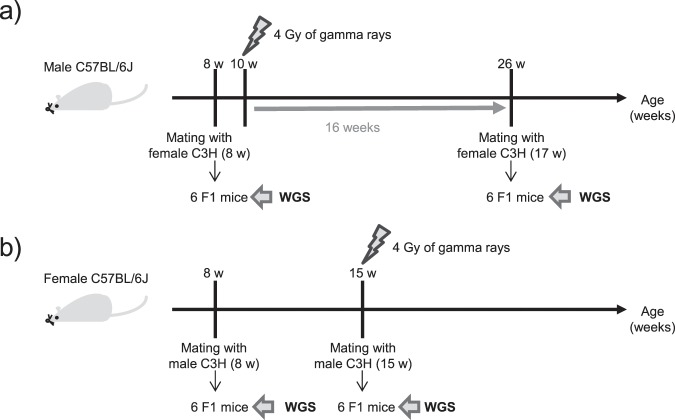
Experimental design of parental exposure. (**a**) Time scheme of the spermatogonia exposure experiment. A male mouse was mated with a female 16 weeks after irradiation. It takes approximately 5 weeks for spermatogonia to develop into sperm during the spermatogenesis process^8^. Thus, the F1 mice born to the exposed father were derived from irradiated spermatogonia. (**b**) Time scheme of the mature oocyte exposure experiment. A female mouse was mated with a male just after irradiation, and thus, the F1 mice born to the exposed mother were derived from irradiated mature oocytes.

In the spermatogonia exposure experiment (Fig. 1a), a non-irradiated male C57BL/6J mouse was mated with a non-irradiated female C3H/HeN mouse to produce F1 mice, which served as controls. Subsequently, the same male was irradiated with 4 Gy of gamma rays. Sixteen weeks after irradiation, the irradiated male was mated with a second non-irradiated female C3H/HeN mouse, which resulted in the generation of F1 mice from the irradiated male. These F1 mice were among the mice included in our previous study examining *de novo* copy number variants with array-based comparative genomic hybridization (array-CGH)^15^. In the mature oocyte exposure experiment (Fig. 1b), a non-irradiated female C57BL/6J mouse was mated with a non-irradiated male C3H/HeN mouse to produce F1 mice, which served as controls. After the offspring were weaned, the female was subsequently exposed to 4 Gy of gamma rays. Immediately afterward, the irradiated female mouse was mated with the same non-irradiated C3H/HeN male and gave birth to F1 mice. In this experiment, the birth date of the offspring indicated that the exposed mature oocytes were fertilized at midnight following the day of irradiation.

A total of 24 F1 mice (six F1 mice born before and six F1 mice born after irradiation in the spermatogonia and mature oocyte exposure experiments) and their parents were subjected to WGS with at least 33× total read coverage (Supplementary Data 1). The raw sequence data were mapped to the mouse C57BL/6J reference sequence (mm10), and *de novo* mutations were called using HaplotypeCaller (Genome Analysis Toolkit). To achieve an accurate comparison of the *de novo* mutations between the groups, we focused on a specific autosomal genomic region (the effective whole-genome coverage [EWC] region^16^, see Methods), and high-quality sequences of all the nucleotides in this region were obtained from the WGS of all the mice in the spermatogonia and oocyte exposure experiments. This region corresponds to 53.9% of the entire mouse autosomal genome (1,290,348,172 bp) and includes 70.5% of all autosomal exon regions.

For the 24 F1 mice, we obtained a total of 336 *de novo* SNV candidates and 79 *de novo* indel candidates in the EWC region (Supplementary Data 2). Indels occurring in repeat sequences that consisted of more than a certain number of repeat units were excluded from our *de novo* variant list (see Methods) because they were difficult to detect precisely. In addition, we defined any cluster of mutations (SNVs and indels) with a shared haplotype within 100 base pairs (most of the mutations occurred within 15 bp) as a multisite mutation. In this study, a multisite mutation was counted as a single mutation separate from SNVs and indels. A subsequent validation experiment using Sanger sequencing showed highly reliable results, which indicated that all the tested candidates (53 SNVs and 43 indels) were successfully confirmed as *de novo* variants in the F1 mice with the exception of three SNV candidates that could not be amplified by PCR.

### Number of *de novo* mutations detected in the offspring

The numbers of *de novo* mutations in the offspring born before and after the parental exposures are shown in Table 1. First, using the number of mutations detected in the F1 mice born before irradiation (mating at 8 weeks of age), we estimated the spontaneous per-generation mutation rate. The SNV rate was estimated to be 4.0 × 10^−9^ (95% confidence interval = 3.3–4.7 × 10^−9^) per nucleotide, and no significant difference in the pre-irradiation estimates was found between the spermatogonia and oocyte exposure experiments (*P* = 0.17, based on a two-tailed statistical test using Poisson simulation; see Methods). This rate is comparable to a previous estimate in laboratory mice (3.8 × 10^−9^ per nucleotide per generation; for parents aged 8 weeks at conception)^7^ and somewhat lower than the rate of 5.4 × 10^−9^ (95% confidence interval = 4.6–6.5 × 10^−9^) mutations per nucleotide per generation reported for a mean parental age of 12 weeks at conception^16^. For indels, the spontaneous per-generation mutation rate (assuming mating at 8 weeks of age) was estimated as 3.9 × 10^−10^ (95% confidence interval = 2.1–5.7 × 10^−10^) per nucleotide, and the estimates from the paternal and maternal exposure experiments were not significantly different (*P* = 0.66, according to a two-tailed test based on Poisson simulation). Although the values cannot be easily compared due to the different detection conditions, particularly the method used to address variants in repeat sequences (Methods), the indel rate was similar to our previous estimate of 3.1 × 10^−10^ (95% confidence interval = 1.2–6.4 × 10^−10^) per nucleotide per generation (given a mean parental age of 12 weeks at conception)^16^.

**Table 1:** Number of *de novo* mutations in the offspring before and after exposure to IR.

Group	No. of mutations^a^	Mutation rate (95% CI)	*P*^b^	No. of mutations adjusted for parental age^c^	*P* ^d^
*SNVs*					
Spermatogonia exposure					
Before IR	70	4.5 × 10^−9^ (3.5–5.7 × 10^−9^)		70	
After IR	128	8.3 × 10^−9^ (6.9–9.8 × 10^−9^)	0.0001	**65**.**1**	0.91
Mature oocyte exposure					
Before IR	54	3.5 × 10^−9^ (2.6–4.6 × 10^−9^)		54	
After IR	84	5.4 × 10^−9^ (4.3–6.7 × 10^−9^)	0.012	**54**.**6**	0.96
*Indels*					
Spermatogonia exposure					
Before IR	5	3.2 × 10^−10^ (1.0–7.5 × 10^−10^)		5	
After IR	42	2.7 × 10^−9^ (2.0–3.7 × 10^−9^)	<0.00001	**35**.**9**	<0.00001
Mature oocyte exposure					
Before IR	7	4.5 × 10^−10^ (1.8–9.3 × 10^−10^)		7	
After IR	25	1.6 × 10^−9^ (1.0–2.4 × 10^−9^)	0.0022	**22**.**1**	0.0095
*Multisite mutations*					
Spermatogonia exposure					
Before IR	2	1.3 × 10^−10^ (0.16–4.6 × 10^−10^)		2	
After IR	10	6.5 × 10^−10^ (0.31–1.2 × 10^−9^)	0.031	10	0.031
Mature oocyte exposure					
Before IR	0	0 (0–2.4 × 10^−10^)		0	
After IR	10	6.5 × 10^−10^ (0.31–1.2 × 10^−9^)	0.0031	10	0.0031

^a^The numbers of mutations in the EWC region identified in six F1 mice per group are shown.

^b^The probability of a difference between before and after IR was estimated using a two-tailed test based on Poisson simulation of the difference.

^c^The numbers of mutations after adjusting for parental age effects (with 8 weeks as the reference age) in six F1 mice are shown, and the adjusted values are shown in bold.

^d^The probability of a difference between before and after IR was estimated using a two-tailed test. To estimate the probability of SNVs and indels, the uncertainty of aging effects on *de novo* SNVs and indels was incorporated in similar simulations using Poisson and, in some cases, binomial random variables.

We subsequently compared the number of *de novo* mutations between the F1 mouse groups born before and after irradiation. In both the spermatogonia and mature oocyte exposure experiments, the offspring born after parental irradiation exhibited higher numbers of *de novo* SNVs, indels, and multisite mutations compared with those found in the offspring born prior to irradiation (Table 1). This increase could be explained by two possible causes: one possibility is that parental radiation exposure could induce *de novo* mutations in the genome of the offspring, and the other possibility is that the parental age at conception could affect the number of mutations in the offspring born (Fig. 1). Recent human trio WGS studies have suggested that an advanced parental age at conception increases the numbers of *de novo* SNVs and indels in the offspring^17–21^. Therefore, it is necessary to discriminate whether these increases in mutations are due to the effect of irradiation or the effect of parental aging.

### Effects of radiation and parental aging on mutations

At present, the quantitative relationship between parental aging in mice and increases in *de novo* mutations is unknown. To evaluate the effects on spontaneous *de novo* SNVs in mice, we compared the number of mutations that occurred in the unexposed parental allele (e.g., the maternal allele in the spermatogonia exposure experiment) between offspring born from parents aged 8 weeks (before irradiation) and offspring born later (after irradiation) (Fig. 1). Using nearby polymorphic sites between the fathers and mothers (one belonging to the C57BL/6J strain and the other belonging to the C3H/HeN strain), we identified the parental origins of 94 SNVs (27.9% of the observed SNVs) that occurred on a paternal or a maternal allele (Supplementary Data 3A). A comparison of the number of *de novo* variants on the unexposed parental alleles indicated that an amount equivalent to 4.5% (paternal) and 2.3% (maternal) of the total number of *de novo* SNVs observed in the offspring of parents aged 8 weeks at conception would be added each week (Supplementary Data 3B). These estimates are not very reliable because the number of phased SNVs was limited. Therefore, we used another approach to confirm this aging effect. By comparing the two aforementioned estimates of spontaneous SNV mutation rates in mice (5.4 × 10^−9^ per nucleotide for a parental age of 12 weeks at conception [previous study]^16^ and 4.0 × 10^−9^ per nucleotide for a parental age of 8 weeks at conception [current study], both datasets were obtained using fundamentally the same method), we obtained another estimate for the effect of parental aging as an 8.8% increase in *de novo* SNVs per week (an estimate that included paternal and maternal effects). Because the estimates from two independent methods appeared to be close, we used a 6.8% increase (paternal: 4.5% and maternal: 2.3%) per week in the current analysis.

According to this estimate (Supplementary Data 4), the number of *de novo* mutations adjusted for parental aging effects is shown in Table 1. Most of the increases in SNV counts observed in offspring of the exposed parents in both the spermatogonia and mature oocyte exposure experiments could be explained by these aging effects. We cannot exclude the possibility that radiation exposure induces some SNVs, but it is unlikely that parental radiation exposure of 4 Gy would induce a number of SNVs comparable to spontaneous mutations. This finding is similar to a previous result that showed that the irradiation of 3 Gy of mouse sperm does not increase the number of SNVs in the offspring^7^.

For *de novo* indels and multisite mutations, the parental aging effect on the number of *de novo* mutations is more difficult to evaluate than that on SNVs due to a lack of previous data and a shortage of phased variants. However, in both paternal and maternal exposure experiments, the number of *de novo* mutations in the offspring born to irradiated parents was substantially higher than that in the offspring born to pre-irradiation parents (Table 1). The numbers of these types of mutations are not known to increase rapidly with increases in parental age, even in previous human studies (~2.0 SNVs, ~0.1 indels and ~0.0 multisite mutations added per year of increase in parental age)^20,22^, and most of the changes between the offspring from before and after exposure appear to be due to parental irradiation.

Although the increase in the absolute number of indels is thought to be small, we estimated the parental aging effect on indels to evaluate the effects of radiation exposure. According to a human study, the ratio of indels to SNVs is nearly constant, regardless of the parental age at conception^19^. This finding suggests that the ratio of the age-associated increase in the number of mutations to the number of spontaneous mutations is similar between SNVs and indels. Therefore, we used the value found for the aging effects of SNVs (6.8% increase per week [paternal: 4.5% and maternal: 2.3%]) in our evaluation of the aging effects of indels (Supplementary Data 4). The number of *de novo* indels adjusted for this parental aging effect is shown in Table 1. Compared with the controls (offspring born to unexposed parents), significant increases in the numbers of *de novo* indels were found in the offspring from the father exposed at the spermatogonia stage (*P* < 0.00001, based on a two-tailed statistical test using Poisson-based simulation, Supplementary Figure) and in the offspring from the exposed mother (*P* = 0.0095). Assuming that the parental aging effect is two-fold greater than our estimates of SNVs, the increase in indels after irradiation was still significant (*P* = 0.0012 for the spermatogonia exposure experiment and *P* = 0.011 for the mature oocyte exposure experiment). In addition, the age-adjusted number of indels in the offspring of the father with irradiated spermatogonia was somewhat greater than that in the offspring of the mother whose mature oocytes were exposed (*P* = 0.058, a two-tailed test by Poisson-based simulation).

**Figure 2:**
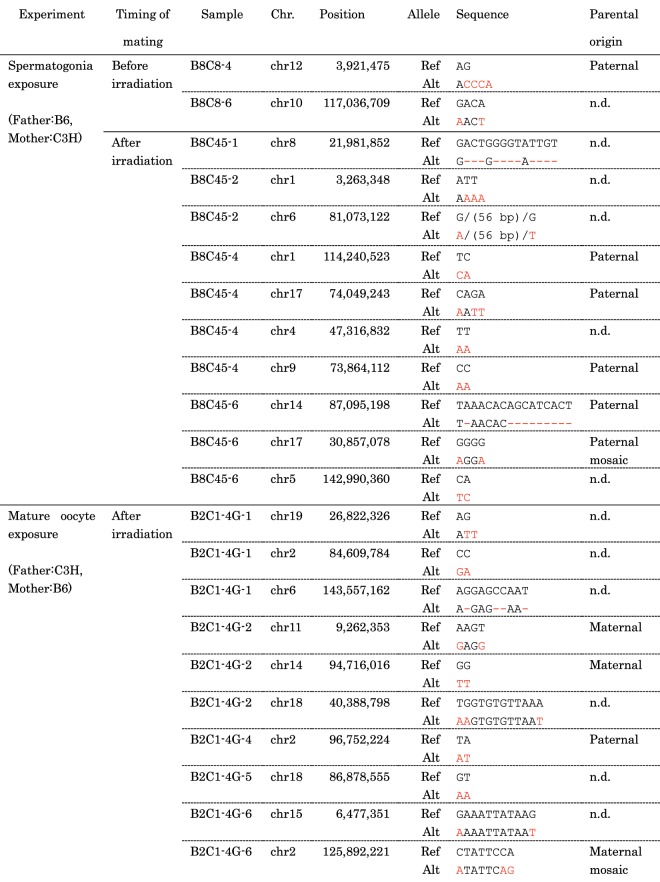
*De novo* multisite mutations detected in this study. The sequences of multisite mutations (Alt) compared with the reference sequence (ref.) are shown in red. The dashed lines show deletions. There is no strain difference of the sequences between B6 and C3H where multisite mutations were occurred. n.d., the parental alleles were not determined due to lack of the neighbouring polymorphism between B6 and C3H.

The number of *de novo* multisite mutations was also significantly higher in the offspring of the irradiated parents. According to a human study^22^, multisite mutations (focusing on an inter-mutational distances <100 bp) occurred at a very low frequency, ~0.07 *de novo* variants per diploid per generation, and no significant effect of parental aging at conception was observed. Therefore, we assumed that parental aging has no effects on *de novo* multisite mutations in mice and therefore assumed that multisite mutations are induced by parental irradiation in both the spermatogonia and mature oocyte exposure experiments (*P* = 0.031 for the spermatogonia exposure experiment and *P* = 0.0031 for the mature oocyte exposure experiment) (Table 1 and Supplementary Figure). Consistent with this view, many multisite mutations were observed in the alleles derived from exposed parents (Fig. 2).

### Characteristics of mutations induced by irradiation

**Figure 3:**
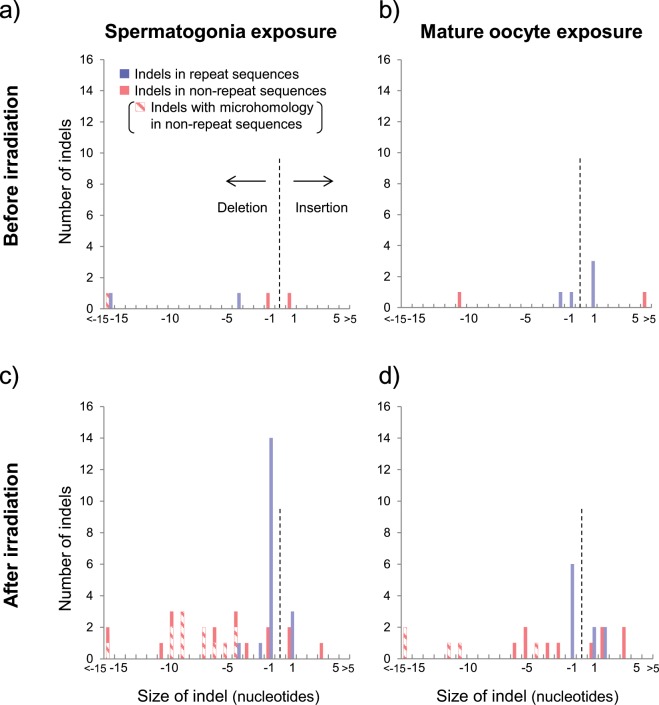
Size distribution of *de novo* indel mutations in F1 mice. The “size of indels” (insertions and deletions) indicates the number of nucleotides inserted (negative values represent deletions). (**a**) Before the exposure of spermatogonia to radiation. (**b**) Before the exposure of mature oocytes to radiation. (**c**) After the exposure of spermatogonia to radiation. (**d**) After the exposure of mature oocytes to radiation. The blue bar shows the number of indels occurring in repeat sequences, and the red bar shows the number of indels occurring in non-repeat sequences. The shaded red bar shows the number of non-repeat sequence indels having microhomology at the breakpoint junction.

To ascertain the features of radiation-associated mutations, we first examined the spectra of *de novo* indels. The sizes of *de novo* indels for deletions and insertions ranged from 1 to 35 and from 1 to 14 nucleotides, respectively (Fig. 3). We compared the number of mutations by adjusting for the parental age effects between F1 mice born before and after parental irradiation. We found 7.7-fold increase in the number of deletions in the offspring of the father with exposed spermatogonia (*P* < 0.00001, based on a two-tailed statistical test using Poisson-based simulation) and 4.7-fold increase of that in the offspring of the mother with exposed mature oocytes (*P* = 0.014) compared with the controls (offspring born to unexposed parents), whereas no significant increase in the number of insertions in the offspring of the exposed father (*P* = 0.13) and mother (*P* = 0.19) was observed. We also noted that the number of deletions in the offspring of the exposed father was significantly higher than that in the offspring of the exposed mother (*P* = 0.0053, a two-tailed test by Poisson-based simulation). These results indicated that deletions were the main type of mutation and that the number of *de novo* deletions might depend on the germ cell stage at the time of exposure.

Regarding the deletions, we frequently found two types of mutations in the offspring of the irradiated parents (Fig. 3 and Supplementary Data 5). One mutation type is single-nucleotide deletions occurring in mononucleotide repeat sequences. More than half of the single-nucleotide deletions were located in repeat sequences consisting of two successive nucleotides, such as a deletion from AA to A (Table 2). The other mutation type is deletions (1 ~ 35 nucleotides) occurring in non-repeat sequences. Approximately 60% of non-repeat sequence deletions in the range of 3 ~ 35 nucleotides showed microhomology (2 ~ 4 nucleotides) at the breakpoint junction (12 out of 18 with spermatogonia exposure and 5 out of 9 with mature oocyte exposure). Both types of deletions were increased in the offspring of irradiated parents (spermatogonia exposure, *P* = 0.00018 for single-nucleotide deletions and *P* < 0.00001 for non-repeat sequence deletions; mature oocyte exposure, *P* = 0.089 for single-nucleotide deletions and *P* = 0.0047 for non-repeat sequence deletions; a two-tailed test by Poisson-based simulation). In addition, both types of deletions appeared at a higher frequency in the offspring of the exposed father than in the offspring of the exposed mother (*P* = 0.061 for single-nucleotide deletions and *P* = 0.045 for non-repeat sequence deletions, a two-tailed test by Poisson-based simulation).

**Table 2:** Breakdown of single nucleotide indels in mononucleotide repeats.

Experiment	Timing of mating	Length of mononucleotide repeats^a^	No. of indels^b^	
			Deletion (−1 base)	Insertion (+1 base)
Spermatogonia exposure	Before irradiation	Non-repeat	1	1
		2	0	0
		3	0	0
		4–7	0	0
	After irradiation	Non-repeat	2	2
		2	8	0
		3	3	1
		4–7	3	2
Mature oocyte exposure	Before irradiation	Non-repeat	0	0
		2	0	1
		3	1	0
		4–7	0	2
	After irradiation	Non-repeat	0	1
		2	4	2
		3	1	0
		4–7	1	0

Single nucleotide insertions and deletions are classified as repeat-associated mutations and non-repeat mutations.

^a^The “length of mononucleotide repeats” represents the number of units of successive mononucleotide sequences in which the mutations occurred. For example, a “length of mononucleotide” equal to 3 refers to sequences such as AAA, CCC, GGG and TTT. Single nucleotide indels occurring in mononucleotide repeats with a length greater than 7 were excluded from this study (see Methods).

^b^The numbers of indels identified in six F1 mice per group are shown.

**Figure 4:**
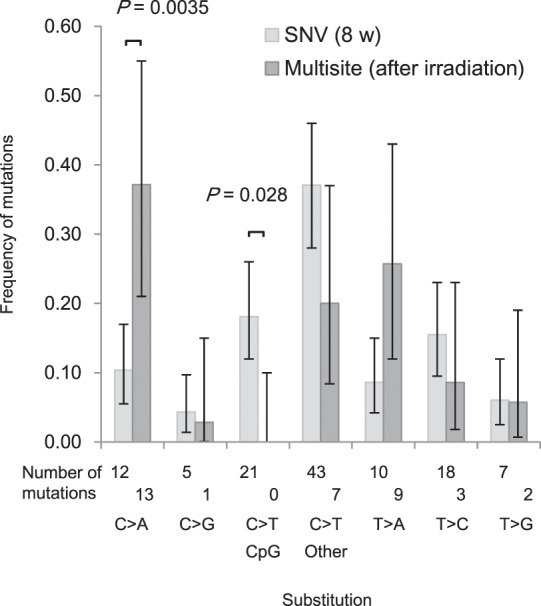
Profile of spontaneous SNVs and nucleotide substitutions present within multisite mutations. We used SNVs observed in a total of 12 F1 mice whose parents were mated at 8 weeks of age as a reference for spontaneous germline SNVs. For multiple sites, we used nucleotide substitutions within the multisite mutations detected in a total of 12 F1 mice born after parental irradiation. The significance of the differences was estimated using a two-tailed Fisher’s exact test. Error bars, binomial 95% CI. *P* values were obtained after Bonferroni correction.

In most of the multisite mutations, two changes occurred within 15 nucleotides (Fig. 2). Out of a total of 20 multisite mutations in the offspring of exposed parents, eight variants showed successive dinucleotide substitutions. All of these variants were different from the known characteristic dinucleotide substitutions GC > AA and GA > TT, which are thought to be caused by error-prone polymerase^23^. We also found that the spectrum of base substitutions within a multisite mutation was different from the spectrum of common germline *de novo* base substitutions in mice (Fig. 4). C > T substitutions at CpG sites, which are derived from the deamination of methylated C sites, were not detected in the multisite mutations (*P* = 0.028 by Bonferroni-corrected Fisher’s exact test). In contrast, C > A substitutions were increased in the multisite mutations compared with the common germline base substitutions (*P* = 0.0035 by Bonferroni-corrected Fisher’s exact test). C > A substitution is a well-known feature derived from reactive oxygen species, such as 8-oxoguanine^24^.

We also considered the functional impact of the induced mutations. Out of the 67 indels and 20 multisite mutations observed in the offspring born to exposed parents, three indels (~4.5%) were identified in exonic regions, constituting 3.5% of the EWC region; 20 indels (~30%) and nine multisite mutations (~45%) were found in intronic regions, constituting 38% of the EWC region; and 44 indels (~66%) and 11 multisite mutations (~55%) were located in intergenic regions, constituting 58% of the EWC region. A significant difference in mutation occurrence was not observed among regions (*P* = 0.41 for indels and *P* = 0.58 for multisite mutations by a Chi-square test). These findings indicated that IR would not induce mutations exclusively in specific functional regions. A single nucleotide deletion identified in the offspring of the exposed mother caused a frameshift of the *Eps8l3* gene, whose homozygous knockouts show a craniofacial phenotype^25^, but caused no apparent abnormal phenotype. Among the 12 F1 mice in the spermatogonia exposure experiment, one mouse that was born to the irradiated father had a 4,967 kb deletion that we identified in our previous study using array CGH^15^. On the chromosome with the large deletion, only one multisite mutation was detected.

## Discussion

The nature of the mutations induced in the entire genome of germ cells by radiation exposure has long been an important issue in the field of radiation biology. Here, we conducted a WGS analysis and assessed relatively small mutations (mainly up to a few base pairs in size). Extrapolation of our results to regions outside of the EWC region indicated that paternal exposure (spermatogonia) to 4 Gy induced 9.6 indels, most of which were deletions, and 2.5 multisite mutations in the autosomes of each F1 mouse and that maternal exposure (mature oocytes) to 4 Gy induced 4.7 indels and 3.1 multisite mutations per F1 mouse (Table 3). The mutations assessed in this study do not include large-size indels (more than 40 bases) or a large portion of tandem repeat site indels because it was difficult to detect these with high accuracy. The induction of indels and multisite mutations was observed in a previous study^7^, which showed that 1.9 indels and 1.5 multisite mutations were induced by paternal exposure to 3 Gy at the sperm stage. However, whether the apparent differences in the numbers of mutations between the sperm exposure study and our current study are meaningful remains unclear because the mutation detection conditions, particularly the indel detection conditions, used in the sperm study were substantially different from those used in our study.

**Table 3:** Number of IR-induced mutations per mouse offspring.

Timing at exposure	Dose	No. of *de novo* mutations per F1 mouse^a^ (95% CI)			Reference
		SNVs	Indels	Multisite mutations	
Spontaneous mutations (at 8 w)^b^	—	19 (16, 23)	1.9 (0.96, 3.2)	0.31 (0.04, 1.1)	This study
Spermatogonia	4 Gy (gamma rays)	n.d.^c^	+9.6 (+5.3, +14.2)	+2.5 (+0.03, +4.5)	This study
Mature oocytes	4 Gy (gamma rays)	n.d.	+4.7 (+1.5, +8.0)	+3.1 (+0.9, +5.0)	This study
Sperm ^d^	3 Gy (X-rays)	n.d.	[+1.9]	[+1.5]	Adewoye *et al*.^7^

^a^The total number of mutations in all autosomes per offspring mouse was estimated through extrapolation of our results to outside of the EWC region. The 95% CI obtained assuming a Poisson distribution is given in brackets. To calculate the 95% CI of indels, the uncertainty of the aging effects on *de novo* indels was incorporated in simulations using Poisson and, in some cases, binomial random variables.

^b^The number of *de novo* spontaneous mutations was estimated from the presented results for the offspring born to parents mated at 8 weeks of age (unexposed control).

^c^Not determined at that value. No significant increase in the number of SNVs was found.

^d^The estimated numbers of indels and multisite mutations induced by the irradiation of sperm, as reported in a previous study (Adewoye *et al*.^7^), as shown. Note that it is difficult to directly compare the values to our data because the conditions used for mutation detection (particularly indel detection) differed between the two studies. According to our calculation, the number of spontaneous mutations per F1 mouse (parental age of 8 weeks) in the previous study^7^ was estimated as 18 SNVs, 1.3 indels, and 0.19 multisite mutations, and these values are comparable to our data (19 SNVs, 1.9 indels, and 0.31 multisite mutations).

Many prior studies have indicated that 4 Gy of gamma rays leads to approximately 160 double-stranded DNA breaks, more than 500 clustered lesions, and more than 10,000 other instances of DNA damage (single-strand breaks and base damage) in the mammalian genome^2,26^. The fact that the number of mutations detected in these WGS studies (~10 *de novo* mutations) is notably smaller than the number of such estimated damaged sites supports the contemporary view that almost all sites damaged in spermatogonia, sperm cells, and mature oocytes would be correctly repaired^2^. Presumably, most of the damaged sites in sperm cells and some of the damaged sites in mature oocytes would be repaired in fertilized eggs^9^. After such repair activity, a very small portion of the damage is inherited as a *de novo* mutation in the genome of the offspring.

To our knowledge, this report is the first to show the spectrum of small size indels induced genome-widely by IR in mammalian germlines. We observed two types of deletions: deletions (main range: 1~12 nucleotides) occurred in the non-repeat sequences and single-nucleotide deletions occurred in mononucleotide repeat sequences (Fig. 3). Many of these deletions showed microhomology (2~4 nucleotide homology) or repeat sequence at the breakpoint junction, which indicated that these mutations were derived from the nonhomologous end-joining repair pathway of double-strand breaks. Our finding that more than half of the single-nucleotide deletions occurred in two-base-long mononucleotide repeat sites, such as AA and GG (Table 2), raises the possibility that the strand breaks in such repeat sequences are difficult to be repaired without mutation induction. Mononucleotide repeat deletions have also been observed in previous studies using mice harbouring reporter genes which revealed IR-associated mutation signatures^27–29^. In addition, these features of deletions are consistent with the results of a previous WGS study examining secondary malignancies after radiotherapy^30^, which showed that small deletions exhibiting microhomology at the breakpoint junction and single nucleotide deletions in the mononucleotide repeat sequence frequently occurred in the radiation-associated tumours. These deletions might be a typical signature of mutations induced by irradiation in mammalian cells.

Consistent with a previous study^7^ showing the effect of radiation exposure on mouse sperm cells, the induction of multisite mutations was observed in both the spermatogonia and mature oocyte exposure experiments. Multisite mutations are believed to result from clustered lesions caused by IR^26,31^. A recent human study indicated that *de novo* multisite mutations were increased in the offspring of fathers who worked with radar units^32^. Considered in this context, a multisite mutation is also a potential signature of mammalian germline mutations induced by IR.

In contrast to indels and multisite mutations, we found no significant increase in SNVs derived from IR in either the spermatogonia or the mature oocyte exposure experiment (Table 1), consistent with the results of a previous sperm exposure experiment^7^. Our results suggest that the number of *de novo* SNVs is associated with the parental age at conception rather than the effect of IR. Further studies will be necessary to characterize in detail the effects of IR on SNVs.

We found a significant difference in the age-adjusted number of small-size deletions between spermatogonia and mature oocyte exposure (*P* = 0.0053, a two-tailed test by Poisson-based simulation). Higher frequencies of both types of deletions (deletions in non-repeat sequences and deletions in mononucleotide repeat sites) were observed in the spermatogonia exposure experiment than in the mature oocyte exposure experiment (Fig. 3). The total number of deletions induced by IR in the spermatogonia exposure experiment was 2.2-fold higher than that found in the mature oocyte exposure experiment. The tendency to obtain increased frequencies of deletions after paternal irradiation was not observed for multisite mutations. This inconsistency might be due to differences in the mutagenesis mechanisms: the deletions are likely to originate from double-strand breaks, and multisite mutations are likely obtained from cluster lesions caused by IR. Although cluster damage is thought to occur essentially at the time of irradiation, strand breaks can also occur in response to phenomena other than irradiation (e.g., cell proliferation). Therefore, one possible explanation is that the additional deletions observed in the spermatogonia exposure experiment is derived from the compensatory cell proliferation that occurred after the high degree of spermatogonial stem cell death induced by exposure to 4-Gy radiation^33,34^.

In this current study, we assessed only relatively small mutations (mainly up to a few base pairs in size) due to technical limitations. Previous studies using array-CGH indicated that the exposure of mouse male germ cells to 3~4-Gy radiation induced *de novo* copy number variants (CNVs), mainly large deletions up to a mega base scale, in the offspring^7,15^ and that the frequency of induced CNVs obtained after paternal exposures at the spermatogonia and sperm stages is similar. In addition, according to a previous study^3^ using a mouse specific-locus test, parental radiation exposure increases the number of mutations, which disrupts specific gene loci, in the offspring, and this effect depends on the dose. The exposure to 3-Gy radiation at the sperm stage induced twice as many mutations as irradiation at the spermatogonia stage^11^. The exposure of maturing or mature oocytes to 3~4-Gy radiation also induced twice as many mutations as the irradiation of spermatogonia cells^3^. These data suggested that the timing at which germ cells are exposed to IR can induce higher frequencies of some types of mutations. It is possible that cell competition among germ cells might impact the selective exclusion of some large-scale mutations. Future research is needed to uncover the effects of IR exposure at various germ cell stages on various types of mutations, and improvements in WGS technology, including long-read sequencing techniques, will make it easier to analyse wider ranges of mutations.

Our observations showed that both spermatogonia and mature oocyte exposure (4 Gy) would induce several small mutations in mouse offspring. It is known that there are differences in radiation sensitivity between human and mouse. For example, 1 Gy or less is the threshold of immature oocyte sensitivity to be sterile in mice, but about 5 Gy for humans^12,35^. Therefore, it doesn’t mean that the mutations found in the mouse experiment are directly induced in humans. In addition, the effect of these mutations on the phenotype of the offspring remains obscure. In this study, we found no phenotypic effects in the 12 offspring mice born to the exposed parents. Previous phenotype-based studies on human populations (offspring of exposed people such as atomic bomb survivors) have not shown clear heritable effects of parental exposure to IR^3–5^. It remains controversial whether the frequency of minisatellite mutations is increased in the human offspring of irradiated parents^36,37^. In the near future, the picture of the effect of IR at the whole-genome level will become clearer and more comprehensive. The knowledge about the genome-wide effects of IR would provide new insights into the results of phenotype-based studies such as large-scale cohort studies on IR-exposed human populations.

## Methods

### Mice

All animal experiments were approved by the Institutional Animal Care and Use Committee of the National Institute of Radiological Sciences (NIRS: Chiba, Japan) (Approval No. 10-1006) and by the Experimental Animal Care Committee of the Radiation Effects Research Foundation (RERF: Hiroshima, Japan) (Approval No. RP 2-13). They were performed in accordance with the Guidelines for Animal Experiments of the NIRS and with the Guidelines for Animal Experiments of the RERF. We used two groups of mice (Fig. 1). The first group of mice were used in the male exposure experiment, in which radiation was delivered at the spermatogonia stage. These mice were previously examined with array-CGH^15^. F1 mice were derived from the cross of a C57BL/6J male (sire’s ID, B8^15^) and C3H/HeN females (dams’ IDs, C8 and C45^15^) before and after irradiation with 4 Gy of ^137^Cs gamma rays (Gammacell; Nordion Inc., Ottawa, Canada) at a dose rate of 0.5 Gy/min at the NIRS. The male mouse “B8” at 8 weeks of age was mated with the female mouse “C8” at 8 weeks of age and was then exposed to IR at 10 weeks of age. After 16 weeks, the “B8” mouse, which was 26 weeks of age, was mated with the female mouse “C45” at 17 weeks of age. The second group of mice was used for the female exposure experiment, in which mature oocytes were irradiated. At 8 weeks of age, a female C57BL/6J mouse was mated with a male C3H/HeN mouse to produce F1 mice before irradiation. Once the female mouse reached 15 weeks of age, the animal was exposed to gamma rays at 4 Gy. Later that day, the irradiated female was mated with the same C3H/HeN male mouse. F1 mice obtained from this mating were used for subsequent experiments as the offspring produced after irradiation.

### WGS analysis

High-molecular-weight genomic DNA was extracted from the spleen, liver, and kidneys through the proteinase K/phenol extraction method. Spleen DNA samples from F1 and parental mice were used for WGS, and the remaining DNA was used for confirmation of the mutation candidates. The extracted DNA solutions were used to prepare paired-end libraries according to the user manual of the Illumina sample preparation kit, either with PCR amplification (spermatogonia exposure experiment) or without PCR amplification (mature oocyte exposure experiment). The libraries were sequenced on the Illumina HiSeq 4000 (spermatogonia exposure experiment) or HiSeq X (mature oocyte exposure experiment) platform using 150 bp paired-end reads. The average total read coverage among the samples was 35 reads per nucleotide for the spermatogonia exposure experiment and 49 reads per nucleotide for the mature oocyte exposure experiment (Supplementary Data 1). The sequence reads were mapped to a mouse reference genome (mm10) using the BWA-MEM algorithm^38^. PCR duplicates were removed using Picard (http://broadinstitute.github.io/picard).

### Variant calling

We used mapped sequence data from autosomes to call variants. To decrease the number of false variant calls, we used only high-mapping-quality reads from the mapped autosomal data, i.e., those reads with a mapping quality of 60 (using SAMtools^39^, samtools view –q 60 –f 0 × 2 − F 0 × 500), and hard-clip and soft-clip reads were excluded from variant calling. Variants that were different from the mouse reference genome were called using GATK^40^ HaplotypeCaller to obtain high-mapping-quality reads of mapped autosomal data with a minimum base quality of at least 20. The variant calling of multiple samples consisting of F1 mice and their parents was performed with HaplotypeCaller. Annotations of genomic variants and predictions of their functional effects were conducted using SnpEff^41^.

### Mutation calling and effective whole-genome coverage region

*De novo* mutations were called from variants by filtering with the following inclusion criteria: (1) a variant allele frequency (VAF) less than 0.1 in both parents and (2) a VAF of at least 0.25 in F1 mice. Using these filters, we called *de novo* SNV and small indel candidates, and the *de novo* mutation candidates were reviewed by visual inspection using Integrative Genomics Viewer^42^. For the validation experiment, a subset of the mutation candidates (selected sequentially from the top of the variant list of each F1 individual on chr1, chr2, …) were subjected to Sanger sequencing.

To clearly compare the *de novo* mutations among samples from the different experiments, we defined the EWC region as the region that satisfied the following conditions: i) the lower and upper coverage bounds were set to 50% and 300%, respectively, of the peak coverage of high-mapping-quality reads mapped to autosomal data and ii) the minimum depth ratio of high-mapping-quality reads of mapped autosomal data to all reads of mapped autosomal data at each site was set to 80%. The EWC region was composed of the regions shared by all F1 mice and their parents in both the spermatogonia exposure experiment and the mature oocyte exposure experiment. The *de novo* mutations detected in the EWC region were highly reliable compared with the mutations detected outside of the EWC region. Most mutations in the EWC region exhibited higher variant quality scores, as demonstrated using EAGLE^43^ software.

### Mutation calling for indels

To obtain a credible list of *de novo* indel mutations, we used an additional filter. We divided the indel variants into two groups: indels that occurred in repeat sequences and indels that occurred in non-repeat sequences. We defined “indels that occurred in repeat sequences” as indels that increased (or decreased) the number of tandem repeat units (tandem repeat units were defined as the sites having more than one repeat unit). The called indel variants located in repeat sequences, particularly those consisting of many repeat units, exhibited a particularly high false positive rate. Therefore, we excluded the following indels located in repeat sequences: (1) mutations in mononucleotide repeat sequences having more than 7 units, (2) mutations in dinucleotide repeat sequences having more than 4 units, and (3) mutations in trinucleotide (or more) repeat sequences having more than 2 units of the repeat. To search for deletions that exhibit microhomology, we defined deletions that satisfied the following criteria as microhomologous deletions: (1) the deletion length was at least three nucleotides and (2) two nucleotides of the deleted sequence were identical to the flanking sequences of the deletion.

### Multisite mutations

We defined a multisite mutation as more than one alteration (SNV or indel) occurring within 100 base pairs in the same allele. Multisite mutations were counted separately as mutations different from SNVs and indels. For analysis of the spectra of base substitution within multisite mutations, the condition with the fewest alterations was used for each multisite range. The statistical significance of the spectral difference between the base substitutions within multisite mutations and spontaneous SNVs was assessed in two ways. First, to obtain an entire indication of whether the mutation distribution differed between two groups, we applied Fisher’s exact test comparing the number of mutations and mutation types by group. Second, if there was a significant difference between two groups, we conducted Fisher’s exact test to identify which type of mutations differed. To account for multiple testing, we applied a Bonferroni correction by multiplying the obtained P values by the number of mutation categories (seven in this case).

### Estimation of the mutation rate

The mutation rate *μ* was calculated as *μ* = *m*/(*n* × *G*), where *m* is the total number of mutations, *n* is the number of individual mouse offspring, and *G* is the size of the analysed genome in base pairs (that is, twice the size of the EWC region). In this study, to define the mutation rates of indels and multisite mutations, we counted an occurrence of one indel or one multisite variant as one mutation. The 95% confidence intervals of the mutation rate were calculated using an exact Poisson test in R (poisson.test)^44^.

### Estimation of the effects of radiation and parental aging

We estimated the parental aging effects on *de novo* SNVs by comparing the numbers of mutations of paternal and maternal origin in unexposed alleles before and after exposure (Supplementary Data 3). To determine the parental origins of the mutations, we examined neighbouring polymorphic sites (SNPs or indels) in the father and mother (derived from strain differences between C57BL/6J and C3H). Here, we assumed that there is no strain-specific feature of mutagenesis between C57BL/6J and C3H because we found no difference in the mutation rates and paternal-maternal ratios (eight paternal and eight maternal in the spermatogonia exposure experiment and 10 paternal and seven maternal in the mature oocyte exposure experiment) at 8 weeks of age between the spermatogonia exposure experiment (father; B6, mother; C3H) and the mature oocyte exposure experiment (father; C3H, mother; B6). We also performed linear extrapolation in this aging effect estimation because previous human data showed a linear relationship between the age of the parent at conception and the number of DMNs per offspring^17–21^. After estimating the number of aging-related mutations, we calculated the number of mutations adjusted for parental age by subtracting them from the number of observed mutations after exposure (Supplementary Data 4). To evaluate the effect of radiation exposure on parental germ cells, we compared the number of mutations before exposure with the number of parental age-adjusted mutations after exposure. To estimate the parental age effects on *de novo* indels, we used the same value as that used to estimate the aging effects on SNVs.

### Statistics

We tested the significance of the differences in the numbers of SNVs, indels, and multisite mutations between offspring born before and after parental irradiation using a similar Poisson simulation as described in a previous report^7^ (Supplementary Figure). We conducted 100,000 extractions of mutations according to the Poisson distribution assuming that the numbers of mutations before and after irradiation are the same. We estimated the significance of the differences by calculating the proportions of the 100,000 simulations that gave an excessive number of mutations compared with observed values. We used a two-tailed test for the comparisons of simulated with observed data. We incorporated the uncertainty of the aging effects on *de novo* SNVs and indel mutations in our calculations using the numbers of mutations after adjusting for the parental age effects. In the calculation of multisite mutations, we did not calculate the uncertainty of the aging effects because multisite mutations with an inter-mutational distance of less than 100 bp in humans did not show aging effects^22^, and we assumed that the effects were similar in mice. To calculate the 95% confidence intervals shown in Table 3, we performed 1,000 Poisson-based simulations.

## Supplementary information

Supplementary data.

## Data Availability

The raw sequence data were submitted to the DDBJ Sequence Read Archive (DRA; https://www.ddbj.nig.ac.jp/dra/index-e.html) under Accession Number DRA008165.

## References

[1] Muller HJ (1927). Artificial Transmutation Of The Gene. Science.

[2] Dubrova YE (2016). [Mutation Induction in the Mouse and Human Germline]. Genetika.

[3] Nakamura N, Suyama A, Noda A, Kodama Y (2013). Radiation effects on human heredity. Annu. Rev. Genet..

[4] Little MP, Goodhead DT, Bridges BA, Bouffler SD (2013). Evidence relevant to untargeted and transgenerational effects in the offspring of irradiated parents. Mutat. Res..

[5] Grant EJ (2015). Risk of death among children of atomic bomb survivors after 62 years of follow-up: a cohort study. Lancet Oncol..

[6] Searle AG (1974). Mutation induction in mice. Adv. Radiat. Biol..

[7] Adewoye AB, Lindsay SJ, Dubrova YE, Hurles ME (2015). The genome-wide effects of ionizing radiation on mutation induction in the mammalian germline. Nat. commun..

[8] Adler ID (1996). Comparison of the duration of spermatogenesis between male rodents and humans. Mutat. Res..

[9] Matsuda Y, Tobari I (1989). Repair capacity of fertilized mouse eggs for X-ray damage induced in sperm and mature oocytes. Mutat. Res..

[10] Rathke C, Baarends WM, Awe S, Renkawitz-Pohl R (2014). Chromatin dynamics during spermiogenesis. Biochim. Biophys. Acta.

[11] Russell WL, Bangham JW, Russell LB (1998). Differential response of mouse male germ-cell stages to radiation-induced specific-locus and dominant mutations. Genetics.

[12] Sankaranarayanan K, Nikjoo H (2015). Genome-based, mechanism-driven computational modeling of risks of ionizing radiation: The next frontier in genetic risk estimation?. Mutat. Res. Rev. in Mutat. Res..

[13] Suh EK (2006). p63 protects the female germ line during meiotic arrest. Nature.

[14] By T Staff Of The Jackson Laboratory. Biology of the Laboratory Mouse (ed. Green, E. L.), 2^nd^ ed. McGraw-Hill, New York (1966).

[15] Asakawa JI (2016). Genome-Wide Deletion Screening with the Array CGH Method in Mouse Offspring Derived from Irradiated Spermatogonia Indicates that Mutagenic Responses are Highly Variable among Genes. Radiat. Res..

[16] Uchimura A (2015). Germline mutation rates and the long-term phenotypic effects of mutation accumulation in wild-type laboratory mice and mutator mice. Genome Res..

[17] Kong A (2012). Rate of *de novo* mutations and the importance of father’s age to disease risk. Nature.

[18] Wong WS (2016). New observations on maternal age effect on germline *de novo* mutations. Nat. commun..

[19] Jonsson H (2017). Parental influence on human germline *de novo* mutations in 1,548 trios from Iceland. Nature.

[20] Besenbacher S (2016). Multi-nucleotide *de novo* Mutations in Humans. PLoS Genet..

[21] Turner TN (2017). Genomic Patterns of *de novo* Mutation in Simplex Autism. Cell.

[22] Goldmann JM (2018). Germline *de novo* mutation clusters arise during oocyte aging in genomic regions with high double-strand-break incidence. Nat. Genet..

[23] Harris K, Nielsen R (2014). Error-prone polymerase activity causes multinucleotide mutations in humans. Genome Res..

[24] Ohno M (2014). 8-oxoguanine causes spontaneous *de novo* germline mutations in mice. Sci. Rep..

[25] Dickinson ME (2016). High-throughput discovery of novel developmental phenotypes. Nature.

[26] Sage E, Shikazono N (2017). Radiation-induced clustered DNA lesions: Repair and mutagenesis. Free. Radic. Biol. Med..

[27] Nohmi T (1999). Spi(-) selection: An efficient method to detect gamma-ray-induced deletions in transgenic mice. Env. Mol. Mutagen..

[28] Ono T (1999). Molecular nature of mutations induced by a high dose of x-rays in spleen, liver, and brain of the lacZ-transgenic mouse. Env. Mol. Mutagen..

[29] Masumura K (2002). Heavy-ion-induced mutations in the gpt delta transgenic mouse: comparison of mutation spectra induced by heavy-ion, X-ray, and gamma-ray radiation. Env. Mol. Mutagen..

[30] Behjati S (2016). Mutational signatures of ionizing radiation in second malignancies. Nat. commun..

[31] Eccles LJ, O’Neill P, Lomax ME (2011). Delayed repair of radiation induced clustered DNA damage: friend or foe?. Mutat. Res..

[32] Holtgrewe M (2018). Multisite *de novo* mutations in human offspring after paternal exposure to ionizing radiation. Sci. Rep..

[33] Hasegawa M, Zhang Y, Niibe H, Terry NH, Meistrich ML (1998). Resistance of differentiating spermatogonia to radiation-induced apoptosis and loss in p53-deficient mice. Radiat. Res..

[34] Bastos H (2005). Flow cytometric characterization of viable meiotic and postmeiotic cells by Hoechst 33342 in mouse spermatogenesis. Cytometry Part. A: the J. of the Int. Soc. for Anal. Cytology.

[35] UN Sci. Comn Eff. At. Radiat. Sources, effects and risks of ionizing radiation. *UNSCEAR 1988 REP*., UN, New York.

[36] Dubrova YE (1996). Human minisatellite mutation rate after the Chernobyl accident. Nature.

[37] Kodaira M, Izumi S, Takahashi N, Nakamura N (2004). No evidence of radiation effect on mutation rates at hypervariable minisatellite loci in the germ cells of atomic bomb survivors. Radiat. Res..

[38] Li H, Durbin R (2010). Fast and accurate long-read alignment with Burrows-Wheeler transform. Bioinformatics.

[39] Li H (2009). The Sequence Alignment/Map format and SAMtools. Bioinformatics.

[40] Van der Auwera GA (2013). From FastQ data to high confidence variant calls: the Genome Analysis Toolkit best practices pipeline. Curr. Protoc. in Bioinforma..

[41] Cingolani P (2012). A program for annotating and predicting the effects of single nucleotide polymorphisms, SnpEff: SNPs in the genome of Drosophila melanogaster strain w1118; iso-2; iso-3. Fly.

[42] Robinson JT (2011). Integrative genomics viewer. Nat. Biotechnol..

[43] Kuo T, Frith MC, Sese J, Horton P (2018). EAGLE: Explicit Alternative Genome Likelihood Evaluator. BMC Med Genomics.

[44] Long H, Behringer MG, Williams E, Te R, Lynch M (2016). Similar Mutation Rates but Highly Diverse Mutation Spectra in Ascomycete and Basidiomycete Yeasts. Genome Biol. Evol..

