# Supplementary material for: Characteristics of induced mutations in offspring derived from irradiated mouse spermatogonia and mature oocytes
Source: Sci Rep. 2020 Jan 8;10:37. doi: 10.1038/s41598-019-56881-2 (PMC6949229; doi:10.1038/s41598-019-56881-2)
Supplement: Supplementary file 1 — Supplementary data. [file 41598_2019_56881_MOESM1_ESM.pdf]

Characteristics of induced mutations in offspring derived from irradiated mouse spermatogonia and mature oocytes

Yasunari Satoh[1,*], Jun-ichi Asakawa[1], Mayumi Nishimura[2], Tony Kuo[3,4], Norio Shinkai[3], Harry M. Cullings[5], Yohei Minakuchi[6], Jun Sese[3,4,7], Atsushi Toyoda[6], Yoshiya Shimada[8,9], Nori Nakamura[1], Arikuni Uchimura[1,*]

[1]Department of Molecular Biosciences, Radiation Effects Research Foundation, 5-2 Hijiyama Park, Minami-ku, Hiroshima, 732-0815, Japan
[2]Department of Radiation Effects Research, National Institute of Radiological Sciences (NIRS), National Institutes for Quantum and Radiological Science and Technology (QST), Chiba, 263-8555, Japan
[3]Artificial Intelligence Research Center, AIST, 2-3-26 Aomi, Koto-ku, Tokyo, 135-0064, Japan
[4]Real World Big-Data Computation Open Innovation Laboratory, AIST-Tokyo Tech, 2-12-1 Okayama, Meguro-ku, Tokyo, 152-8550, Japan
[5]Department of Statistics, Radiation Effects Research Foundation, 5-2 Hijiyama Park, Minami-ku, Hiroshima, 732-0815, Japan
[6]Comparative Genomics Laboratory, National Institute of Genetics, Mishima, 411-8540, Japan
[7]Humanome Lab, Inc., L-HUB 3F, 1-4, Shumomiyabi-cho, Sinjuku-ku, Tokyo, 162-0822, Japan
[8]Department of Radiological Sciences, Graduate School of Human Health Sciences, Tokyo Metropolitan University, Tokyo, 116-8551, Japan
[9]Executive Director, QST, Chiba, 263-8555, Japan

*__Corresponding authors__
E-mail: ysatoh@rerf.or.jp
E-mail: uchimura@rerf.or.jp

Supplementary Figure

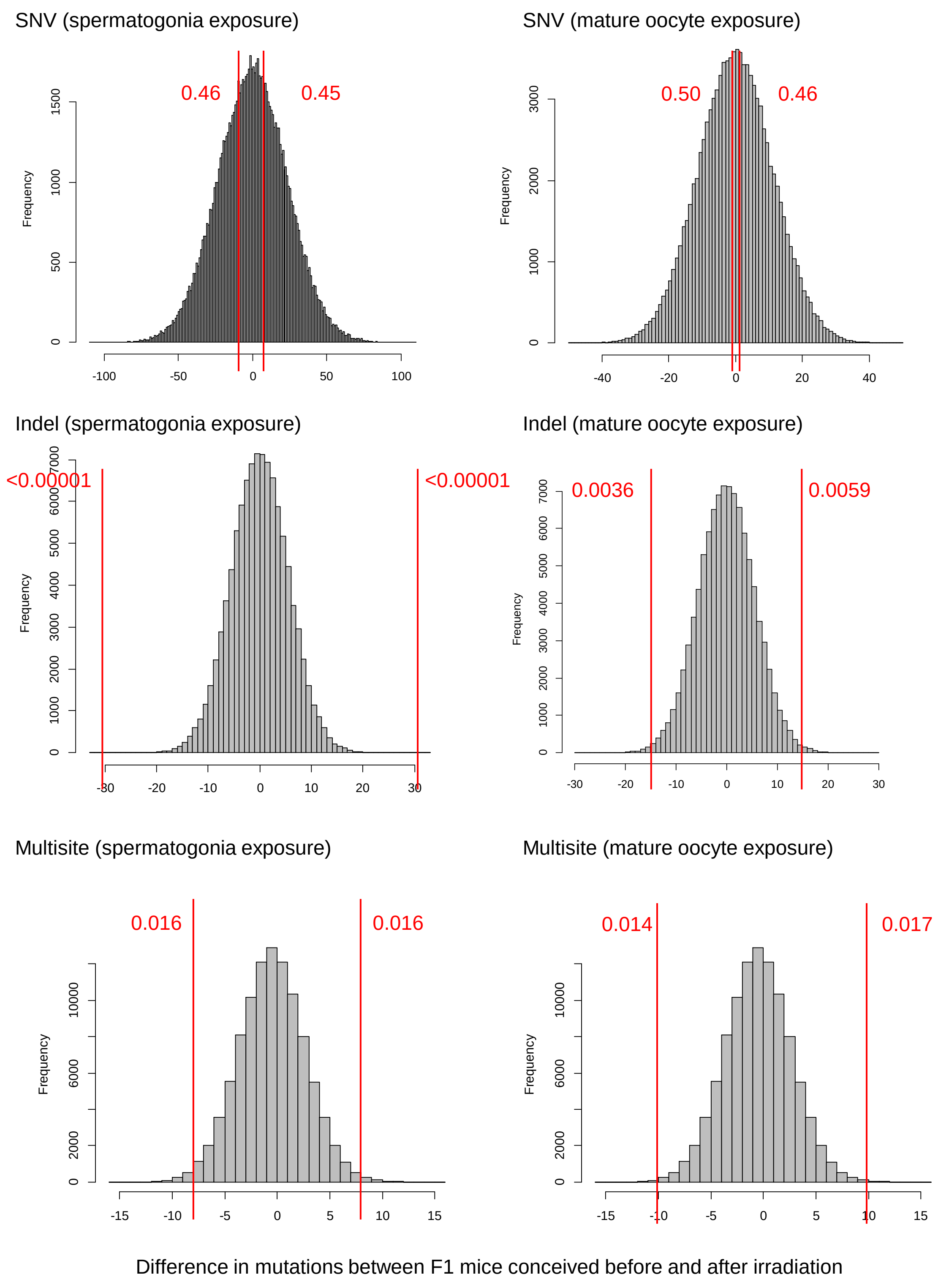

Supplementary Figure. The histogram shows the difference in the number of mutations in F1 mice before and after irradiation obtained after 100,000 mutation extractions assuming a Poisson distribution and that the number of mutations before and after irradiation is the same. To calculate the number of mutations after irradiation, we used the numbers adjusted for the parental age effects with a reference parental age at conception of 8 weeks (Table 1). The uncertainty of the aging effects on *de novo* SNVs and indels was incorporated in simulations using Poisson and binomial random variables. The red lines show the difference in the number of observed mutations before and after irradiation, and the numbers of red-coloured lines show the frequency at which the simulated data values were higher than the observed values.

Supplementary References

1. Asakawa JI, *et al.* Genome-Wide Deletion Screening with the Array CGH Method in Mouse Offspring Derived from Irradiated Spermatogonia Indicates that Mutagenic Responses are Highly Variable among Genes. *Radiat Res* **186**, 568-576 (2016).

Supplementary Data 1. Samples of mouse families and Illumina sequencing data

Sample name	Experiment	Relation	Father	Mother	Timing of mating	Library	Total read coverage (reads per base)	Total reads (all reads mapped autosomal data)	Total bases (all reads mapped autosomal data)	Total bases of HMQ BAM files (High mapping quality reads mapped autosomal data)
B6-8	spermatogonia exposure	Father				Conventional	39	480,980,095	72,147,014,250	61,148,870,550
C3H-8	spermatogonia exposure	Mother				Conventional	33	431,028,232	64,654,234,800	54,013,857,450
C3H-45	spermatogonia exposure	Mother				Conventional	37	469,645,679	70,446,851,850	58,443,570,300
B8C8-1	spermatogonia exposure	F1(female)	B6-8	C3H-8	Before irradiation	Conventional	34	416,610,635	62,491,595,250	50,344,797,150
B8C8-2	spermatogonia exposure	F1(female)	B6-8	C3H-8	Before irradiation	Conventional	34	432,890,256	64,933,538,400	54,484,764,450
B8C8-3	spermatogonia exposure	F1(female)	B6-8	C3H-8	Before irradiation	Conventional	33	465,001,455	69,750,218,250	56,754,960,450
B8C8-4	spermatogonia exposure	F1(female)	B6-8	C3H-8	Before irradiation	Conventional	35	463,135,494	69,470,324,100	57,189,231,600
B8C8-5	spermatogonia exposure	F1(female)	B6-8	C3H-8	Before irradiation	Conventional	34	494,150,445	74,122,566,750	58,350,409,050
B8C8-6	spermatogonia exposure	F1(male)	B6-8	C3H-8	Before irradiation	Conventional	34	476,913,378	71,537,006,700	57,662,758,050
B8C8-7	spermatogonia exposure	F1(male)	B6-8	C3H-8	Before irradiation	Not used in WGS				
B8C45-1	spermatogonia exposure	F1(female)	B6-8	C3H-45	After irradiaiton	Conventional	33	450,323,153	67,548,472,950	56,349,838,650
B8C45-2	spermatogonia exposure	F1(female)	B6-8	C3H-45	After irradiaiton	Conventional	34	447,749,499	67,162,424,850	53,194,188,150
B8C45-3	spermatogonia exposure	F1(female)	B6-8	C3H-45	After irradiaiton	Conventional	36	434,359,435	65,153,915,250	54,322,241,550
B8C45-4	spermatogonia exposure	F1(female)	B6-8	C3H-45	After irradiaiton	Conventional	34	422,001,342	63,300,201,300	53,229,170,850
B8C45-5	spermatogonia exposure	F1(female)	B6-8	C3H-45	After irradiaiton	Conventional	34	472,410,683	70,861,602,450	58,363,417,800
B8C45-6	spermatogonia exposure	F1(male)	B6-8	C3H-45	After irradiaiton	Conventional	34	472,429,280	70,864,392,000	57,980,858,700
B6-2	Mature oocyte exposure	Mother				PCR-free	55	742,201,687	111,330,253,050	93,952,114,800
C3H-1	Mature oocyte exposure	Father				PCR-free	46	644,546,811	96,682,021,650	78,996,743,250
B2C1-C1	Mature oocyte exposure	F1(female)	C3H-1	B6-2	Before irradiation	PCR-free	54	569,056,294	85,358,444,100	70,662,487,500
B2C1-C2	Mature oocyte exposure	F1(female)	C3H-1	B6-2	Before irradiation	PCR-free	56	579,174,382	86,876,157,300	73,306,462,200
B2C1-C3	Mature oocyte exposure	F1(female)	C3H-1	B6-2	Before irradiation	PCR-free	48	672,683,536	100,902,530,400	84,846,898,200
B2C1-C4	Mature oocyte exposure	F1(male)	C3H-1	B6-2	Before irradiation	PCR-free	53	561,003,821	84,150,573,150	69,964,170,150
B2C1-C5	Mature oocyte exposure	F1(male)	C3H-1	B6-2	Before irradiation	PCR-free	36	505,364,300	75,804,645,000	62,686,263,750
B2C1-C6	Mature oocyte exposure	F1(male)	C3H-1	B6-2	Before irradiation	PCR-free	46	649,652,376	97,447,856,400	80,648,334,450
B2C1-C7	Mature oocyte exposure	F1(male)	C3H-1	B6-2	Before irradiation	Not used in WGS				
B2C1-C8	Mature oocyte exposure	F1(male)	C3H-1	B6-2	Before irradiation	Not used in WGS				
B2C1-4G-1	Mature oocyte exposure	F1(female)	C3H-1	B6-2	After irradiaiton	PCR-free	46	621,838,323	93,275,748,450	78,356,214,750
B2C1-4G-2	Mature oocyte exposure	F1(female)	C3H-1	B6-2	After irradiaiton	PCR-free	46	646,929,580	97,039,437,000	81,308,474,550
B2C1-4G-3	Mature oocyte exposure	F1(male)	C3H-1	B6-2	After irradiaiton	PCR-free	47	669,830,712	100,474,606,800	84,732,876,600
B2C1-4G-4	Mature oocyte exposure	F1(male)	C3H-1	B6-2	After irradiaiton	PCR-free	56	575,080,065	86,262,009,750	74,174,527,050
B2C1-4G-5	Mature oocyte exposure	F1(male)	C3H-1	B6-2	After irradiaiton	PCR-free	48	678,648,328	101,797,249,200	83,328,932,250
B2C1-4G-6	Mature oocyte exposure	F1(male)	C3H-1	B6-2	After irradiaiton	PCR-free	45	641,044,087	96,156,613,050	79,052,606,550

Supplementary Data 2. The number of *de novo* mutations in each F1 individual

Sample name	Experiment	Timing of mating	Number of *de novo* mutations 1)			Number of mutations that aging effects were adjusted as all mice at 8 weeks of age 2)	
			SNVs	Indels	Multisite mutations	SNVs	Indels
B8C8-1	spermatogonia exposure	Before irradiation	14	2	0	14	2
B8C8-2	spermatogonia exposure	Before irradiation	12	1	0	12	1
B8C8-3	spermatogonia exposure	Before irradiation	16	0	0	16	0
B8C8-4	spermatogonia exposure	Before irradiation	7	0	1	7	0
B8C8-5	spermatogonia exposure	Before irradiation	9	1	0	9	1
B8C8-6	spermatogonia exposure	Before irradiation	12	1	1	12	1
		Sub total	70	5	2	70	5
B8C45-1	spermatogonia exposure	After irradiation	22	6	1	**11.2**	**5.1**
B8C45-2	spermatogonia exposure	After irradiation	24	9	2	**12.2**	**7.7**
B8C45-3	spermatogonia exposure	After irradiation	23	0	0	**11.7**	**0.0**
B8C45-4 3)	spermatogonia exposure	After irradiation	12	7	4	**6.1**	**6.0**
B8C45-5	spermatogonia exposure	After irradiation	18	6	0	**9.2**	**5.1**
B8C45-6	spermatogonia exposure	After irradiation	29	14	3	**14.7**	**12.0**
		Sub total	128	42	10	**65.1**	**35.9**
B2C1-C1	Mature oocyte exposure	Before irradiation	11	0	0	11	0
B2C1-C2	Mature oocyte exposure	Before irradiation	8	2	0	8	2
B2C1-C3	Mature oocyte exposure	Before irradiation	11	2	0	11	2
B2C1-C4	Mature oocyte exposure	Before irradiation	9	1	0	9	1
B2C1-C5	Mature oocyte exposure	Before irradiation	9	1	0	9	1
B2C1-C6	Mature oocyte exposure	Before irradiation	6	1	0	6	1
		Sub total	54	7	0	54	7
B2C1-4G-1	Mature oocyte exposure	After irradiation	18	5	3	**11.7**	**4.4**
B2C1-4G-2	Mature oocyte exposure	After iradiation	14	2	3	**9.1**	**1.8**
B2C1-4G-3	Mature oocyte exposure	After iradiation	16	1	0	**10.4**	**0.9**
B2C1-4G-4	Mature oocyte exposure	After iradiation	8	4	1	**5.2**	**3.5**
B2C1-4G-5	Mature oocyte exposure	After iradiation	19	5	1	**12.4**	**4.4**
B2C1-4G-6 4)	Mature oocyte exposure	After iradiation	9	8	2	**5.9**	**7.1**
		Sub total	84	25	10	**54.6**	**22.1**

1) All mutations were identified in EWC region.
2) Numbers of mutations shown as bold were adjusted for age effects with 8 weeks as the reference age.
3) This mouse had a large deletion (4,967 kb) that we identified in our previous study (Supple. ref. 1) using array CGH.
4) This mouse had a frameshift of the *Eps8l3* gene.

Supplementary Data 3A. Estimation of parental origin of *de novo* SNVs

	Parental age at mating (weeks)		No. of F1	No. of total SNVs	No. of phased SNVs	Estimated No. of parental origin (/ a F1 mouse)		
	Father	Mother				Total	Paternal	Maternal
Control (before IR)	8	8	12	124	33 (Paternal: 18, Maternal: 15)	10.3	5.6	4.7
Spermatogonia exposure (before IR)	8	8	6	70	16 (Paternal: 8, Maternal: 8)			
Mature oocyte exposure (before IR)	8	8	6	54	17 (Paternal: 10, Maternal: 7)			
Spermatogonia exposure (after IR)	26	17	6	128	37 (Paternal: 25, Maternal: 12)	21.3	14.4	6.9
Mature oocyte exposure (after IR)	15	15	6	84	24 (Paternal: 15, Maternal: 9)	14	8.8	5.3

Supplementary Data 3B. Estimated effects of parental aging on the number of *de novo* SNVs (per week)

	No. of increase of mutations (/ week)	Percentage of increase in the number of mutations per week to the number of spontaneous mutations in F1 of 8 week-old parents
Paternal effect	0.46 (=[8.8-5.6]/7) [-0.063, 0.99] 1)	4.5% (= 0.46 / 10.3)
Maternal effect	0.24 (=[6.9-4.7]/9) [-0.18, 0.70]	2.3% (= 0.24 / 10.3)

1) 95% CI was calculated by 1,000 times of simulations based on Poisson distribution and binominal distribution.

Supplementary Data 4. Estimation of the number of *de novo* mutations adjusted for parental aging effects

	Experiment	Mutations per a F1 mouse at 8 weeks of age	Passed weeks of father after 8 weeks of age	Passed weeks of mother after 8 weeks of age	Ratio of weekly increase in the number of mutations (due to paternal aging effect) to the number of spontaneous mutations in 8 weeks of age	Ratio of weekly increase in the number of mutations (due to maternal aging effect) to the number of spontaneous mutations in 8 weeks of age	Estimated number of paternal aging-related increased mutations per a F1 mouse	Estimated number of maternal aging-related increased mutations per a F1 mouse	Estimated number of parental aging-related increased mutations per 6 F1 mouse	Number of observed mutations after IR per 6 F1 mouse	Number of mutations adjusted for parental age per 6 F1 mouse
SNVs	spermatogonia exposure (after IR)	10.3	18	9	0.045	0.023	8.34	2.13	62.9	128	65.1 [10,135] [1)]
	Mature oocyte exposure (after IR)	10.3	7	7	0.045	0.023	3.24	1.66	29.4	84	54.6 [18, 90]
Indels	spermatogonia exposure (after IR)	1	18	9	0.045	0.023	0.81	0.207	6.1	42	35.9 [22, 51]
	Mature oocyte exposure (after IR)	1	7	7	0.045	0.023	0.315	0.161	2.9	25	22.1 [12, 33]

1) 95% CI was calculated by 1,000 times of simulations based on Poisson distribution and binominal distribution.

Supplementary Data 5. The number of *de novo* insertions and deletions in each F1 individual

Sample name	Experiment	Timing of mating	No. of indels	No. of insertions	Insertions identified in repeat sequences	Insertions identified in non-repeat sequences	No. of deletions	1 bp deletions identified in repeat sequences	>1bp deletions identified in repeat sequences	Deletions identified in non-repeat sequences
B8C8-1	spermatogonia exposure	Before irradiation	2	0	0	0	2	0	1	1
B8C8-2	spermatogonia exposure	Before irradiation	1	0	0	0	1	0	0	1
B8C8-3	spermatogonia exposure	Before irradiation	0	0	0	0	0	0	0	0
B8C8-4	spermatogonia exposure	Before irradiation	0	0	0	0	0	0	0	0
B8C8-5	spermatogonia exposure	Before irradiation	1	1	0	1	0	0	0	0
B8C8-6	spermatogonia exposure	Before irradiation	1	0	0	0	1	0	1	0
B8C45-1	spermatogonia exposure	After irradiation	6	1	0	1	5	2	0	3
B8C45-2	spermatogonia exposure	After irradiation	9	1	0	1	8	5	1	2
B8C45-3	spermatogonia exposure	After irradiation	0	0	0	0	0	0	0	0
B8C45-4	spermatogonia exposure	After irradiation	7	2	1	1	5	1	1	3
B8C45-5	spermatogonia exposure	After irradiation	6	1	1	0	5	3	0	2
B8C45-6	spermatogonia exposure	After irradiation	14	1	1	0	13	3	0	10
B2C1-C1	Mature oocyte exposure	Before irradiation	0	0	0	0	0	0	0	0
B2C1-C2	Mature oocyte exposure	Before irradiation	2	1	1	0	1	1	0	0
B2C1-C3	Mature oocyte exposure	Before irradiation	2	2	1	1	0	0	0	0
B2C1-C4	Mature oocyte exposure	Before irradiation	1	1	1	0	0	0	0	0
B2C1-C5	Mature oocyte exposure	Before irradiation	1	0	0	0	1	0	0	1
B2C1-C6	Mature oocyte exposure	Before irradiation	1	0	0	0	1	0	1	0
B2C1-4G-1	Mature oocyte exposure	After irradiation	5	1	1	0	4	1	0	3
B2C1-4G-2	Mature oocyte exposure	After irradiation	2	0	0	0	2	0	0	2
B2C1-4G-3	Mature oocyte exposure	After irradiation	1	0	0	0	1	1	0	0
B2C1-4G-4	Mature oocyte exposure	After irradiation	4	2	0	2	2	1	0	1
B2C1-4G-5	Mature oocyte exposure	After irradiation	5	2	2	0	3	0	0	3
B2C1-4G-6	Mature oocyte exposure	After irradiation	8	4	1	3	4	3	0	1
